# Case Report: Adult proximal humeral aneurysmal bone cyst: radical resection and reconstruction with osteoconductive allograft & reverse arthroplasty—Ecuador's first reported case and functional outcomes

**DOI:** 10.3389/fsurg.2025.1704393

**Published:** 2025-11-04

**Authors:** Gabriel Gamecho Arteaga, Henry Hernández, Chrystian X. Mestanza, Jaime Zurita, Marlon Arias-Intriago, Juan S. Izquierdo-Condoy

**Affiliations:** 1 Departamento de Traumatología, Hospital de Especialidades “Carlos Andrade Marín”, Quito, Ecuador; 2 Pathology Department, SOLCA Quito, Quito, Ecuador; 3 One Health Research Group, Universidad de las Américas, Quito, Ecuador

**Keywords:** aneurysmal bone cyst, proximal humerus, reverse shoulder arthroplasty, osteoconductive allograft, case report

## Abstract

**Introduction:**

Aneurysmal bone cysts (ABCs) are rare benign lesions, with adult cases being exceptionally uncommon. This report details a multidisciplinary approach for managing a proximal humeral ABC in an adult, emphasizing functional restoration.

**Case presentation:**

A 36-year-old male presented with progressive pain, swelling, and restricted shoulder mobility. Imaging revealed an expansile lytic lesion with “soap-bubble” morphology. Histopathology confirmed a primary ABC. After failed conservative management, radical *en bloc* resection was performed, followed by single-stage reconstruction using osteoconductive allograft, reverse shoulder arthroplasty, and rotator cuff repair.

**Conclusion:**

This case demonstrates the efficacy of aggressive, single-stage reconstruction in adult ABCs, combining advanced orthopedic and oncologic principles. It advocates for molecular diagnostics to confirm primary lesions and supports hybrid graft-prosthesis strategies to optimize outcomes. The success of this approach provides a framework for managing rare, large ABCs in adults, encouraging further exploration of tailored surgical innovations in orthopedic oncology.

## 1 Introduction

Aneurysmal bone cysts (ABCs) are rare, benign, yet locally aggressive lesions predominantly affecting adolescents and young adults (1, 2). Characterized by rapid growth and potential for cortical destruction, ABCs account for approximately 0.14 cases per 100,000 individuals annually, with fewer than 25% occurring in patients over 30 years old (3). While histologically benign, their expansive nature can lead to severe functional impairment, particularly when involving weight-bearing or anatomically complex regions such as the proximal humerus (1, 3).

Conventional management of ABCs relies on intralesional curettage with adjuvant therapies, yet recurrence rates remain high (15%–30%), necessitating repeated interventions. In adults, extensive lesions pose unique challenges due to reduced regenerative capacity and the demand for durable structural reconstruction (4, 5). Proximal humeral involvement further complicates treatment, as preservation of shoulder mechanics and rotator cuff integrity becomes critical to restoring upper limb functionality.

This case report describes Ecuador's first documented use of a proximal humeral allograft for reconstruction following aneurysmal bone cyst (ABC) resection. While allograft procedures have been previously reported in other anatomical locations, this represents the first application specifically in the proximal humerus within our national context. Additionally, this case stands among the earliest reported instances of ABC in an adult patient in Ecuador. Our findings provide valuable insights into the surgical management of these rare, anatomically challenging lesions in adults, with particular emphasis on achieving durable recurrence control while maximizing functional restoration.

## 2 Case presentation

**Figure 1:**
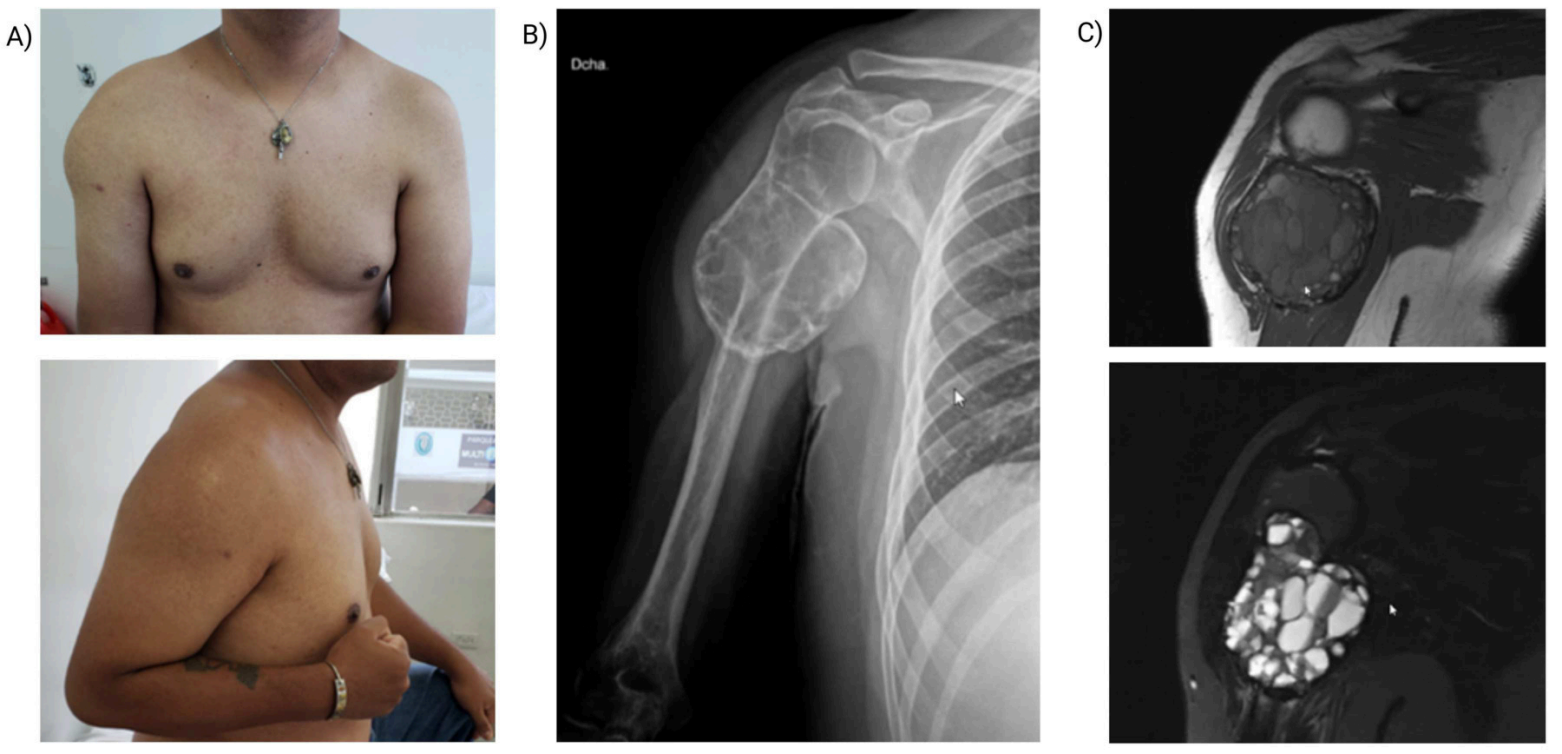
Preoperative patient evaluation. **(A)** Clinical image showing soft tissue displacement at the right proximal humerus. **(B)** Anteroposterior radiograph reveals a bony lesion in the posterolateral metaphysis of the proximal humerus with a bulging periosteal reaction, internal septations, and a soap-bubble appearance, characteristic of aneurysmal bone cyst (ABC). **(C)** Coronal MRI: T1-weighted image (top) shows soft tissue displacement and inflammatory changes in the deltoid muscle. T2-weighted image (bottom) demonstrates fluid levels within the lesion, consistent with ABC.

A 36-year-old man presented with an 8-month history of a progressively enlarging, tender mass in the right proximal humerus after an impact with a chlorine container. He had attempted traditional therapies, including consultation with a sobador (bonesetter), without improvement. Over the preceding month, he reported worsening pain, further enlargement of the mass, and marked functional decline. His medical history was notable for a penicillin allergy. On examination, there was localized edema and chronic tenderness with severely restricted active range of motion (ROM) of the right shoulder; flexion and abduction were each limited to 10°. Initial radiographs demonstrated an irregular cystic lesion of bone, prompting a provisional diagnosis of a neoplasm of uncertain behavior (Figure 1). Analgesics were initiated, and a core needle (Tru-Cut) biopsy was performed.

Pathology results were delayed by 5 months because of resource limitations. The biopsy showed nonspecific findings—fibroblastic proliferation, erythrocyte extravasation, hemosiderin deposits, and multinucleated giant cells—and was deemed inconclusive. The case was referred to a tertiary oncology center for further evaluation. Reassessment of the histologic slides at the specialized center supported a benign aneurysmal bone cyst (ABC); although molecular testing was unavailable in our setting, we recommend performing it when feasible. The patient was subsequently referred back to his base hospital for definitive management.

**Figure 2:**
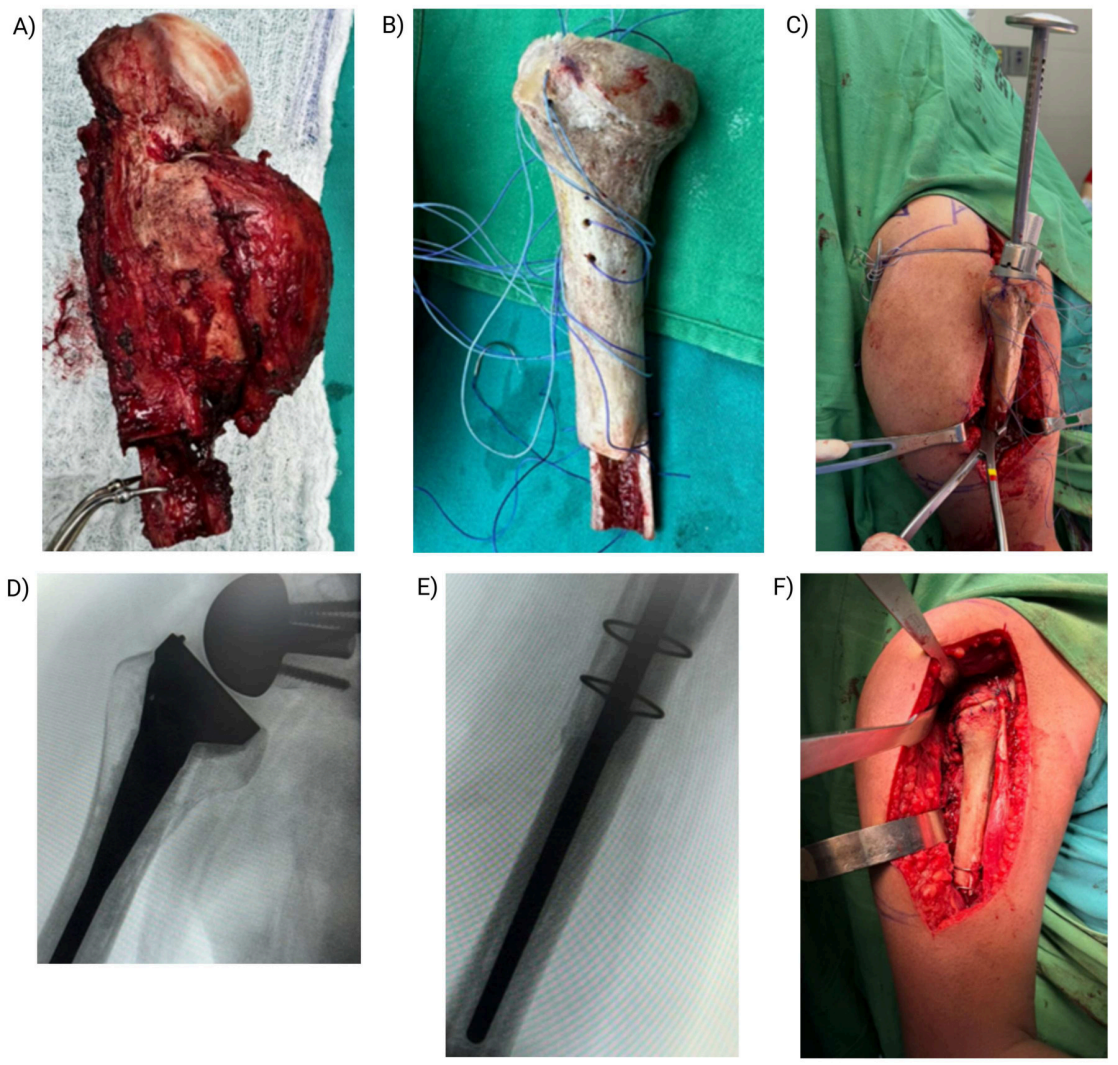
Surgical procedure. **(A)** Resected tumor specimen extending from the surgical neck to the mid-diaphysis. **(B)** Allograft proximal humerus prepared with high-strength sutures for muscle reattachment. **(C)** Implantation of the prosthesis with reattached musculature. **(D,E)** Intraoperative fluoroscopy confirming prosthesis positioning and osteotomies with cerclage wire stabilization. **(F)** Final reconstruction with reverse shoulder prosthesis and reattached musculature.

Six months after initial presentation, he underwent resection of the right proximal humerus lesion with reverse total shoulder arthroplasty and structural bone grafting (allograft–prosthesis composite). The intraoperative and immediate postoperative courses were uncomplicated (Figure 2). Empiric intravenous cephalosporins (avoiding penicillin-based agents due to allergy) were administered for leukocytosis (20.83 × 10^3^/µL), presumed reactive. Early postoperative rehabilitation achieved passive flexion of 60° and abduction of 50°.

Definitive histopathology of the resected specimen confirmed the diagnosis, demonstrating a cyst wall lined by bland squamous epithelium and a hemorrhagic stroma with fibroblastic septa, consistent with ABC. En bloc resection with clear margins was achieved, followed by reconstruction using a structural allograft. The allograft was processed and preserved according to international standards for cold-chain storage and sterile handling to maintain biological safety and structural integrity. No exogenous osteoinductive agents were used; graft incorporation relied on the intrinsic biological response elicited by the surgical procedure. Precise osteotomies and careful preservation of surrounding soft tissues facilitated the release of endogenous osteogenic factors at the host–graft interface, promoting biological integration.

**Figure 3:**
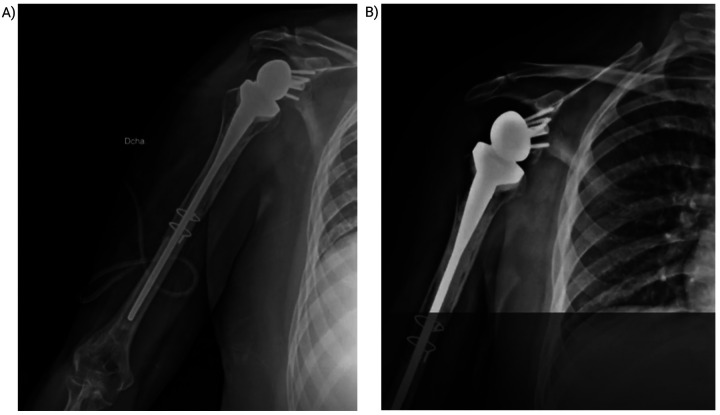
Postoperative control and follow-up. **(A)** Immediate postoperative radiograph showing reverse shoulder prosthesis with cerclage wire and “flute-beak” osteotomies. **(B)** Radiograph at 5 months postoperative demonstrating early osteointegration at the graft-host interface.

At the 3-month postoperative visit, the patient showed a well-healed surgical scar, no radiographic evidence of recurrence, and improved ROM. Repeat imaging and functional assessment were scheduled for 3 months later to monitor recovery and exclude recurrence (Figure 3).

**Figure 4:**
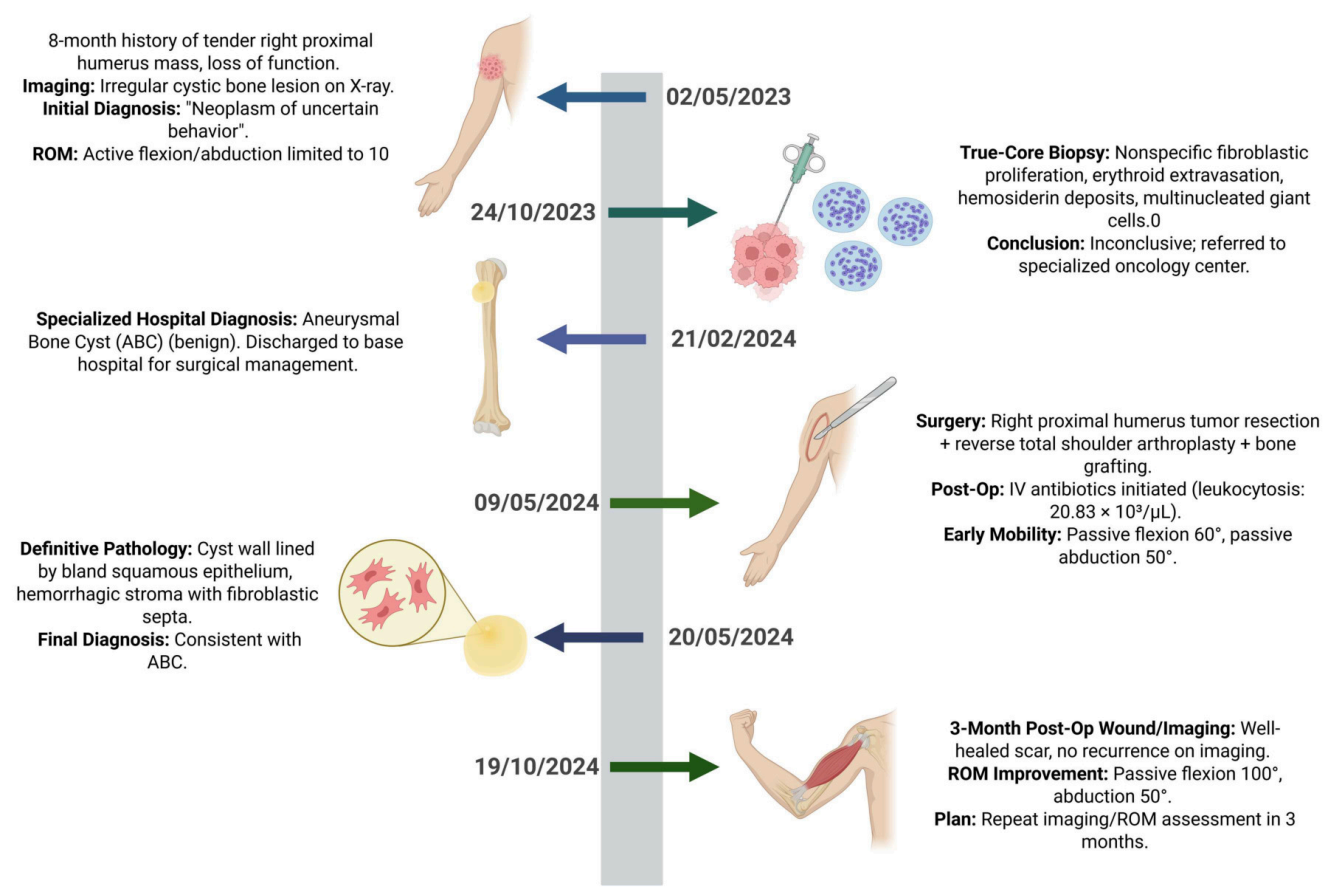
Timeline events.

At the most recent follow-up, the patient reported substantial clinical improvement and enhanced quality of life. Examination of the right shoulder showed: flexion 90°, abduction 90°, adduction 10°, extension 10°, internal rotation 50°, and external rotation 50°. Strength was graded 4/5, sensation 1/2, and deep tendon reflexes 1/4. The surgical site remained clean and dry, without signs of infection or inflammation. Rehabilitation is ongoing, focusing on muscle strengthening, proprioceptive training, and isometric and isotonic exercises to further restore function (Figure 4).

## 3 Discussion

### 3.1 Background

ABC are rare, benign yet locally aggressive lesions, with an estimated annual incidence of 0.14 cases per 100,000 individuals (6). Although approximately 75% of ABCs occur in patients under 20 years of age (1), presentation in adults older than 30 years—as in our case—represents an exceptionally uncommon scenario. In Ecuador, epidemiologic data are scarce, with most reported cases in pediatric populations (7). Despite their benign histology, ABCs may progress rapidly, producing substantial cortical destruction and functional impairment and therefore require timely diagnosis and individualized management (6).

Historically, ABCs were considered tumor-like, reactive processes secondary to vascular or traumatic events that increased venous pressure—an idea that seemed clinically relevant in our patient with prior trauma. However, recent tumor-genetics studies show that ∼70% of ABCs harbor chromosomal translocations, most commonly *t*(16;17)(q22;p13), generating a CDH11–USP6 fusion that upregulates USP6 transcription (3, 8). Notably, USP6 rearrangements are absent in ABC-like changes arising secondarily within other tumor (1).

The most frequent symptoms are pain and swelling—as observed in our patient—and fracture is an uncommon initial event. In the spine, ABCs can compress neural elements and cause neurologic deficits. Although benign, ABCs may result in severe sequelae, underscoring the need for early diagnosis and treatment (1). Clinical behavior is variable, with quiescent, active, and aggressive forms described. The natural history typically spans four phases (6):

- Initial osteolytic phase—cortical destruction and periosteal elevation.
- Active phase—periosteal reaction, neoplastic ossification, and bony expansion.
- Stabilization phase—septation and reduced growth.
- Cicatrization phase—calcification, ossification, and remodeling

In our patient, an initially indolent course was followed by rapid enlargement, marking transition to the active phase, with imaging correlates of aggressive periosteal reaction and cortical thinning.

### 3.2 Imaging

Imaging features vary by evolutionary stage. In our case, radiographs of the proximal humerus showed a lytic, expansile, eccentric lesion with internal trabeculations, cortical thinning, and a “soap-bubble” appearance—classic for ABC (Figure 1) (9, 10). Computed tomography (CT) demonstrated multiloculated cystic spaces with fluid–fluid levels, a hallmark produced by sedimentation of blood products. Magnetic resonance imaging (MRI) confirmed the cystic architecture with heterogeneous T1/T2 signals, enhancing septa, and absence of solid nodular components, effectively arguing against telangiectatic osteosarcoma (3). Although nonspecific, bone scintigraphy highlighted hypervascularity, supporting an active metabolic process (1). Altogether, these findings favored a primary ABC; the principal radiologic differential diagnoses are summarized in Table 1.

**Table 1:** Imaging differential diagnosis of an aneurysmal bone cyst.

Differential diagnosis	Imaging features	Rationale for consideration	Rationale for exclusion	Ref.
Aneurysmal bone cyst (ABC)	Eccentric, expansile lytic lesion with fluid–fluid levels and septations; cortical thinning; no soft-tissue mass	Patient age and imaging pattern compatible with primary ABC	—	(10)
Telangiectatic osteosarcoma	Lytic lesion with cystic areas and fluid–fluid levels but with solid enhancing components and malignant periosteal reaction	Rapid enlargement and overlap with ABC features	Lack of solid nodular areas and absence of a sunburst periosteal reaction.	(11)
Giant cell tumor (GCT)	Expansile lytic lesion at the epiphyseal–metaphyseal junction in skeletally mature patients	Similar location and radiologic appearance	Typically subarticular; fluid–fluid levels uncommon	(12)
Chondroblastoma with secondary ABC changes	Eccentric epiphyseal lesion with chondroid matrix; may show fluid–fluid levels	Can mimic ABC in young adults	Predominantly epiphyseal with matrix calcifications not present here	(13)
Osteoblastoma	Expansile lytic lesion with variable mineralization and surrounding sclerosis	May resemble ABC, especially in spinal posterior elements	No sclerosis or matrix calcification in our lesion	(14)
Non-ossifying fibroma (NOF)	Well-defined, metaphyseal, cortically based lytic lesion with sclerotic rim	Common benign lesion in young people	Usually asymptomatic; lacks expansion and fluid–fluid levels	(15)

### 3.3 Histology

Definitive diagnosis requires correlation of clinical, imaging, and histopathologic findings to distinguish primary from secondary ABC (3). Biopsy—percutaneous or curettage-assisted—is indispensable (6). Grossly, ABCs are well-demarcated, multiloculated masses with blood-filled cystic spaces separated by gritty, tan-white septa, imparting a spongiform appearance (1). Microscopically, cysts lack an endothelial lining and are bordered by fibrous septa containing bland fibroblasts, osteoclast-like giant cells, siderophages, and chronic inflammatory cells. Mitoses may be present without atypia, and reactive osteogenesis appears as delicate woven osteoid or mature trabeculae rimmed by osteoblasts. A calcified basophilic fibrochondroid matrix is present in roughly one-third of cases (1, 3, 6).

Molecular confirmation by FISH can demonstrate USP6 rearrangements at 17p13.2—a defining hallmark of primary ABC that differentiates it from ABC-like changes in GCTs or osteosarcomas (1, 16). These translocations drive USP6-mediated osteoclastic activity and cystic remodeling. Although molecular testing was unavailable in our case due to resource constraints, its incorporation into diagnostic workflows is strongly recommended when feasible.

The principal histologic differential diagnoses are summarized in Table 2, listed from most to least likely, along with distinguishing features.

**Table 2:** Histologic differential diagnosis of an aneurysmal bone cyst.

Differential diagnosis	Key histologic features	Rationale for consideration	Rationale for exclusion	Ref.
Aneurysmal bone cyst (ABC)	Blood-filled cystic spaces without endothelial lining; fibrous septa with fibroblasts, osteoclast-like giant cells, and reactive woven bone; mitoses without atypia	Classic multiloculated appearance with reactive bone formation	—	(1)
Telangiectatic osteosarcoma	Blood-filled spaces with malignant osteoid, pleomorphic cells, and atypical mitoses	Can closely mimic ABC morphologically	Presence of atypical mitoses, malignant osteoid, and cytologic atypia excluded this diagnosis	(11)
Giant cell tumor (GCT) with secondary ABC change	Sheets of mononuclear stromal cells with uniformly distributed giant cells; may show hemorrhagic cystic areas	Common in young adults, may exhibit ABC-like areas	Lack of uniform giant-cell distribution and absence of p63 positivity favored ABC	(17)
Chondroblastoma with secondary ABC	Chondroblasts with “chicken-wire” calcification, scattered giant cells, and possible cystic spaces	Occurs in young adults, may have overlapping features	Absence of chondroblastic differentiation and calcified matrix excluded this	(13)
Giant cell reparative granuloma (GCRG)	Cellular fibroblastic stroma with numerous giant cells and hemosiderin; usually in jaw bones	Histologic overlap with solid variant ABC	Location and lack of cystic spaces made GCRG less likely	(18)
Brown tumor of hyperparathyroidism	Fibroblastic tissue with osteoclast-like giant cells and hemorrhage	May resemble ABC microscopically	Normal serum calcium and PTH levels excluded this reactive lesion	(19)

### 3.4 Management

Nonoperative strategies have gained traction for anatomically challenging or high-risk lesions. These include percutaneous sclerotherapy, embolization, and systemic pharmacologic therapy. Percutaneous sclerotherapy with polidocanol, doxycycline, or ethanol induces endothelial injury and fibrosis within cystic spaces, achieving healing in 70%–90% of selected series (20). Selective arterial embolization—especially valuable for spinal and pelvic lesions—reduces vascularity and may drive gradual regression after staged sessions (21). When surgery is contraindicated or incomplete resection is anticipated, denosumab (a RANKL inhibitor) can suppress osteoclastic activity and shrink cyst volume; nevertheless, prolonged surveillance is essential because recurrence and incomplete ossification remain concerns, particularly in active, expansile lesions that jeopardize structural integrity (22).

Given this patient's rapid progression, cortical destruction, and risk of pathologic fracture, conservative therapy was unsuitable. Surgery was indicated to achieve durable local control, restore stability, and mitigate recurrence risk. Following diagnosis, a 4–6-week interval after biopsy is advisable to allow partial healing or involution before intervention. While wide resection provides the highest likelihood of cure, less aggressive strategies are generally preferred initially, particularly when preservation of structural integrity is paramount (1, 3).

For most ABCs, standard care consists of intralesional curettage with adjuvant measures and bone grafting to reduce recurrence. Allograft is frequently used to fill post-curettage defects, especially in children, where autograft availability is limited. In the series by Olivera Núñez et al., curettage with allograft was associated with a 37.5% recurrence rate, particularly in lesions involving the physis (23). Minimally invasive approaches—such as percutaneous curettage with allogenic bone impaction grafting—have yielded excellent long-term results, with no recurrences after a mean follow-up of 6.4 years (4). For cases requiring structural reconstruction, allograft–prosthesis composites can provide durable stability, including reported reconstructions for proximal humeral ABC (2).

Comparative studies of allograft vs. bioactive glass (BG-S53P4) show no significant difference in recurrence (46% vs. 40%), although bioactive glass may resorb more slowly and enhance remodeling (24). Adjuvant high-speed burring followed by allograft, without chemical adjuvants, has achieved recurrence rates as low as 3.2% (5). For complex or recurrent lesions, endoscopic resection with allograft and platelet-rich plasma (PRP) has shown favorable outcomes, including complete resolution of a calcaneal ABC at 2 years (25). Systematic reviews reinforce that curettage with allograft remains the cornerstone of management, with higher recurrence among younger patients with open physes (23, 26). For spinal or pelvic ABCs, preoperative embolization with subsequent allograft reconstruction may reduce bleeding and complications (2). Ultimately, the choice among autograft, allograft, bioactive glass, and synthetic substitutes should balance mechanical stability, biological integration, and recurrence risk (4, 24).

## 4 Conclusions

This case represents Ecuador's first reported instance of an adult proximal humeral ABC managed through an innovative single-stage reconstruction combining osteoinductive allograft, reverse shoulder arthroplasty, and rotator cuff repair. It provides three pivotal advances in orthopedic oncology: demonstrating the feasibility of integrating biological augmentation with prosthetic replacement for extensive bone loss, establishing molecular diagnosis (USP6 rearrangement) as critical for differentiating primary ABCs from secondary mimics, and challenging the traditional paradigm of conservative management through radical yet functional preservation.

The successful outcome—evidenced by osteointegration, joint stability, and absence of recurrence at follow-up—validates this approach as a viable alternative to conventional staged procedures. Beyond its technical contributions, this case serves as a foundational reference for three emerging concepts: the safety of aggressive resection in selected benign lesions, the potential of hybrid graft-prosthesis constructs in young adults, and the importance of molecular diagnostics in surgical planning. These insights create new therapeutic possibilities for anatomically complex ABCs while setting a precedent for future research into long-term outcomes and expanded applications of combined biological-mechanical reconstruction.

## Data Availability

The original contributions presented in the study are included in the article/Supplementary Material, further inquiries can be directed to the corresponding author.
